# Editorial: The role of electrocardiogram in prediction of cardiovascular and non-cardiovascular health outcomes

**DOI:** 10.3389/fcvm.2025.1694071

**Published:** 2025-09-17

**Authors:** Elsayed Z. Soliman, Dexter Canoy

**Affiliations:** ^1^ Department of Cardiovascular Medicine, Wake Forest University School of Medicine, Winston Salem, NC, United States; ^2^ Faculty of Medical Sciences, Population Health Sciences Institute, Newcastle University, Newcastle, United Kingdom

**Keywords:** electrocardiagram (ECG), ECG markers, artificial intelligence, electrocardiografical disorders, cardiorenal, cardio - pulmonary, cardiac age, heart and brain interaction

**Editorial on the Research Topic**
The role of electrocardiogram in prediction of cardiovascular and non-cardiovascular health outcomes

Electrocardiography (ECG) has been a cornerstone of cardiovascular medicine for more than a century. By capturing the heart's electrical activity through surface electrodes, this deceptively simple test informs clinical decisions across the spectrum of care, from diagnosing acute coronary occlusion and malignant arrhythmias to assessing hypertrophic remodeling and guiding revascularization or pacing strategies. Its simplicity, portability and low cost explain why ECG is ubiquitous in emergency departments, clinics, operating rooms and intensive care units. The Research Topic entitled “*The Role of Electrocardiogram in Prediction of Cardiovascular and non-Cardiovascular Health Outcomes*” brings together eight articles that showcase both the traditional and emerging roles of ECG. In the commentary that follows, we highlight the key themes from these studies and explore future challenges.

## Traditional diagnostic roles of electrocardiography

Several articles in the collection demonstrate the enduring value of ECG in identifying cardiac diseases. Li et al. retrospectively compared 195 hypertrophic cardiomyopathy (HCM) patients with 116 control patients and assessed whether simple voltage parameters could screen for HCM. Peguero S voltage provided the highest sensitivity (70.4%) and combining it with the Sokolow-Lyon index increased sensitivity to 88.7% (Li et al.). A modified Cornell index achieved the highest area under the ROC curve (0.88) for detecting HCM (Li et al.). These findings show that despite advances in imaging, ECG remains a cost-effective screening tool for cardiomyopathies.

The study by Befkadu et al. highlights ECG's value in populations with systemic disease. In their Ethiopian cohort of 96 HIV-infected adults and 96 controls, nearly half of HIV-positive participants showed ECG abnormalities such as ST-segment/T-wave changes and prolonged QT intervals (Befkadu et al.). Smoking, use of protease inhibitors, and low CD4 counts were independently associated with ECG abnormalities, prompting the authors to recommend routine ECG surveillance in patients receiving antiretroviral therapy (Befkadu et al.). Taken together, these reports reinforce the traditional role of ECG as a point-of-care diagnostic tool that detects structural and conduction disorders.

Traditional research applications are also represented. Mostafa et al. used the UK Biobank to explore the relationship between obesity and cardiac conduction defects in over 455,000 participants. They defined conduction defects broadly (atrioventricular or intraventricular blocks) and found that severe obesity increased the odds of composite conduction defect by ∼20% after multivariable adjustment (Mostafa et al.). Each standard deviation increase in body-mass index (4.76 kg m^−2^) was associated with higher odds of atrioventricular block, particularly among older men and people with diabetes (Mostafa et al.). Their discussion links obesity-related epicardial adipose tissue to fibro-fatty infiltration of the atrioventricular node, explaining why obesity preferentially affects atrioventricular rather than intraventricular conduction (Mostafa et al.). This epidemiological study illustrates how large-scale ECG cohorts help uncover modifiable risk factors for conduction disease.

## Beyond disease detection – ECG as a measure of cardiovascular health

Whereas traditional ECG interpretation focuses on diagnosing disease, emerging work uses ECG to quantify cardiovascular health. Ansari et al. leveraged deep learning to derive an “ECG-based heart age” (ECG-age) from short 12-lead recordings. In healthy individuals, ECG-age approximated chronological age; in those with cardiovascular disease (CVD), ECG-age exceeded chronological age, and a large positive delta (ECG-age minus chronological age) predicted worse cardiovascular outcomes (Ansari et al.). Their study evaluated how sampling frequency, signal duration and data augmentation affected the model's generalization. Higher sampling rates improved accuracy, and a lightweight convolutional network (AttiaNet) maintained performance while reducing computational cost (Ansari et al.). These findings illustrate how ECG can capture “cardiovascular age” and thus health, not just disease.

The distinction between health and disease is important. While an abnormal ECG can signal myocardial infarction or arrhythmia, a normal-looking ECG can still carry information about cardiovascular aging. This concept opens possibilities for using ECG in preventive medicine. It also underscores the need for standardized acquisition parameters and robust training strategies, since models trained on clean datasets may fail in noisy real-world environments.

## Probing pathophysiology with ECG

Two articles delve deeper than detection and prediction by using ECG to understand arrhythmia mechanisms. Barold and Herweg revisit Mobitz type II second-degree atrioventricular block and caution that many clinicians misinterpret 2:1 atrioventricular block as Mobitz II. They note that true Mobitz II requires constant PR intervals before and after a single blocked *P* wave, arises in the His–Purkinje system and often necessitates pacemaker implantation Barold and Herweg. The review highlights how vagal surges and atypical Wenckebach phenomena can mimic Mobitz II, emphasizing that detailed ECG analysis is necessary to avoid overdiagnosis. This educational piece demonstrates ECG's role in refining electrophysiological understanding and ensuring appropriate therapy.

Martinez-Navarro et al. used human-based computer models to investigate how ischemic regions shape ventricular fibrillation (VF) dynamics. By varying the location and extent of simulated ischemia, they showed that regional ischemia facilitates re-entry and that remote ischemia modulates VF frequency and amplitude. The amplitude spectrum area of the ECG stratified VF severity and certain electrode positions (apex–anterior and apex–posterior) were most informative (Martinez-Navarro et al.). Such mechanistic modelling demonstrates how ECG signals can be used to probe arrhythmia pathophysiology and optimize defibrillation strategies.

## Artificial intelligence and expanded horizons

The remaining articles exemplify how artificial intelligence (AI) and advanced analytics extend ECG applications to new domains. Leung et al. applied a deep neural network to UK Biobank ECGs to estimate ECG-age and related the delta age to stroke risk. A 10-year increase in delta age conferred a 22% higher risk of incident stroke and 42% higher risk of accelerated aging (Leung et al.). The authors argue that ECG-age could improve existing stroke prediction tools and might be useful in settings where brain imaging is limited (Leung et al.).

Butler et al. extended AI-based ECG analysis to a non-cardiovascular outcome – preeclampsia. Using modified ResNet networks trained on 10-second, 12-lead ECGs from two medical centers, their model detected preeclampsia with an internal cross-validated area-under-the-curve (AUC) of 0.85 and an external AUC of 0.81 (Butler et al.). The model predicted preeclampsia up to 90 days before clinical diagnosis with AUCs as high as 0.92 and achieved 0.98 AUC for early-onset preeclampsia (Butler et al.). These results suggest that subtle maternal cardiac electrical changes precede hypertensive disorders of pregnancy and that ECG may serve as an inexpensive screening tool in low-resource settings.

Together with the heart-age and stroke studies, these AI-enabled analyses show how ECG can reveal latent biological signatures beyond the human eye's capacity. Yet they also reveal common challenges: models trained on one population may not generalize across devices or ethnicities, and subtle biases may propagate when using AI for clinical decision making.

## Challenges and future directions

Several themes emerge across the collection. First, ethical considerations must accompany AI-driven ECG analyses. Models are often “black boxes,” making it difficult to explain individual predictions; this opacity risks undermining patient trust and could lead to inappropriate clinical use. Researchers must prioritize transparency, external validation, and the avoidance of algorithmic bias. Second, adoption of advanced ECG analytics may widen global health disparities. Access to high-quality ECG devices, reliable internet connectivity and computing infrastructure varies greatly between high-income and low-income countries. Without careful planning, AI tools may benefit wealthy settings while under-served populations remain reliant on traditional ECG interpretation. Building affordable devices, training local clinicians, and creating open-source algorithms can help bridge this gap.

Third, standardization is essential. The studies in this collection used different sampling rates, recording durations and preprocessing methods. To compare results and translate AI models across institutions, common data formats and standardized reporting of ECG acquisition parameters are required. International collaborations such as the PhysioNet challenges have begun this process, but more consensus is needed. Finally, new directions beckon. Combining ECG with other physiological signals (e.g., photoplethysmography, wearable accelerometers) might improve prediction of syncope or sudden death. Integration of ECG-derived biomarkers into electronic health records could enable real-time risk stratification. And as models become more interpretable, they may generate hypotheses about underlying biology, as illustrated by the obesity-CCD study's mechanistic discussion.

## Conclusions

This research collection demonstrates the breadth of modern electrocardiography. Traditional applications such as diagnosing cardiomyopathy, conduction disease or arrhythmias remain indispensable. At the same time, advances in AI allow ECG to quantify cardiovascular health, predict non-cardiac outcomes and illuminate pathophysiological mechanisms. To realize these opportunities while minimizing harm, the ECG community must embrace ethical AI, prioritize equity, and establish standards for data collection and algorithm evaluation. The electrocardiogram, once a simple tracing on paper, is evolving into a rich substrate for precision medicine.

